# A Scoping Review on Topics Related to Well-Being in Later Life in Europe

**DOI:** 10.1093/geroni/igaf122.1903

**Published:** 2025-12-31

**Authors:** Dilare Ecenur Irbık, Bram Vanhoutte

**Affiliations:** Universite Libre de Bruxelles, Brussels, Brussels Hoofdstedelijk Gewest, Belgium; Universite Libre de Bruxelles, Brussels, Brussels Hoofdstedelijk Gewest, Belgium

## Abstract

Ageing policy has evolved over the last decades through consecutive policy frameworks, such as healthy ageing, active ageing, or age-friendly environments. Each framework emphasises different aspects of what “ageing better” means and shifts the policy spotlight on new and often previously neglected aspects, sequentially improving the landscape of policies for later life. Going beyond these emphasised aspects of each framework, this paper aims to detect, from the current research literature, topics of ageing supporting well-being in later life which are missing from these policy frameworks. We used Arksey and O’Malley’s five-stage methodological framework to map the available evidence, reported according to the PRISMA Extension for Scoping Reviews (PRISMA-ScR) Checklist. Three databases were used to identify the peer-reviewed quantitative, qualitative, and mixed-methods empirical articles published between January 2002 and January 2024. Articles were included if they were about older adults (Population), reported practices and/or recommendations that support well-being in later life (Concept) and were conducted in EU Member States (Context). Our search string resulted in 12,206 articles, of which 110 papers were retained for analysis. Results show that topics regarding well-being in later life that are missing in the main ageing policy frameworks are older prisoners, transnational families with older members, COVID-19 and emergency response regarding social isolation of older people, and LGBT+ older people. We believe this scoping review will trace the limits of the current policy frameworks and help us to understand which challenges could become central in future ageing frameworks.

